# Analysis of stranded information using an automated procedure for strand specific RNA sequencing

**DOI:** 10.1186/1471-2164-15-631

**Published:** 2014-07-28

**Authors:** Benjamín Sigurgeirsson, Olof Emanuelsson, Joakim Lundeberg

**Affiliations:** Science for Life Laboratory, School of Biotechnology, Royal Institute of Technology (KTH), Tomtebodavägen 23A, 17165 Solna, Stockholm Sweden

**Keywords:** RNA sequencing, Strand specificity, Ribosomal depletion, Bioinformatics, Antisense RNA

## Abstract

**Background:**

Strand specific RNA sequencing is rapidly replacing conventional cDNA sequencing as an approach for assessing information about the transcriptome. Alongside improved laboratory protocols the development of bioinformatical tools is steadily progressing. In the current procedure the Illumina TruSeq library preparation kit is used, along with additional reagents, to make stranded libraries in an automated fashion which are then sequenced on Illumina HiSeq 2000. By the use of freely available bioinformatical tools we show, through quality metrics, that the protocol is robust and reproducible. We further highlight the practicality of strand specific libraries by comparing expression of strand specific libraries to non-stranded libraries, by looking at known antisense transcription of pseudogenes and by identifying novel transcription. Furthermore, two ribosomal depletion kits, RiboMinus and RiboZero, are compared and two sequence aligners, Tophat2 and STAR, are also compared.

**Results:**

The, non-stranded, Illumina TruSeq kit can be adapted to generate strand specific libraries and can be used to access detailed information on the transcriptome. The RiboZero kit is very effective in removing ribosomal RNA from total RNA and the STAR aligner produces high mapping yield in a short time. Strand specific data gives more detailed and correct results than does non-stranded data as we show when estimating expression values and in assembling transcripts. Even well annotated genomes need improvements and corrections which can be achieved using strand specific data.

**Conclusions:**

Researchers in the field should strive to use strand specific data; it allows for more confidence in the data analysis and is less likely to lead to false conclusions. If faced with analysing non-stranded data, researchers should be well aware of the caveats of that approach.

**Electronic supplementary material:**

The online version of this article (doi:10.1186/1471-2164-15-631) contains supplementary material, which is available to authorized users.

## Background

The transcriptome has long been studied by reverse transcribing single stranded RNA into double stranded cDNA and assessed with assays such as PCR [1, 2], microarrays [3, 4] or massively parrallel sequencing [5, 6]. By assessing gene expression through cDNA the strand information of the RNA is lost. With the advent of many strand specific RNA library preparation protocols increasing number of RNA sequencing experiments are generating stranded RNA sequencing data [7–9]. Without strand information it is difficult to determine correct gene expression from overlapping genes; i.e. genes that have the same location in the genome, at least partly, but are transcribed from opposite strands. Knowing the strand information of the cDNA is essential to determine from which of the overlapping genes the RNA originates from. Such overlapping genes in mammalian genomes, while not frequent, are more common than previously thought [10, 11] and they are widespread in genomes of other species, especially those with small and compact genomes [12].

Increasing exploration of the transcriptome has led to discoveries of multitude of various RNA species [13]. Of particular interest with regards to strand information is antisense RNA (asRNA) which is a transcribed RNA that is complementary, i.e. on the opposite strand, to another gene, usually a protein coding gene. Thus by definition all antisense genes are overlapping genes. The most straightforward regulatory function of asRNA is its ability to hybridize to its existing sense mRNA and hinder translation of that particular mRNA molecule. This, however, is just one function of many and asRNA encompasses many different types of RNA [14]. A relatively newly discovered feature of asRNA is the antisense transcription of pseudogenes [15, 16]. Pseudogenes, evolutionary remnants of gene duplication, were long thought to be silent and non-functional. Still, while prokaryotes rapidly lose pseudogenes from their genomes, complex multicellular animals like mammals often retain their pseudogenes, suggesting evolutionary conservation and thus function. Evidence is now mounting towards various regulatory functions of pseudogenes [17].

A handful of protocols have been published which retain the strand information of the RNA with varying degree of success and labor intensity. In 2009 Parkhomchuk et al. [7] published a strand specific library protocol which has since become popular among such protocols being both relatively simple and effective. The protocol is called *dUTP second strand marking method*, or *dUTP method* for short, and consists of using dUTPs instead of dTTPs during the synthesis of the second strand in the cDNA synthesis step during sample preparation. Then prior to PCR amplification the uracil in the second strand is degraded using Uracil-N-Glycosylase (UNG). With the second strand partly degraded only the first strand is amplified in the subsequent PCR. This particular strand specific protocol was evaluated as superior in terms of simplicity and data quality in a benchmark study of strand specific protocols [18].

In the current study we modulate specific steps in a scalable transcriptome preparation method [19] to combine the strand specific dUTP method [7] and the Illumina TruSeq RNA sample preparation kit (# RS-122-2001) into an automated strand specific RNA sequencing protocol. By preparing libraries from different cancer cell lines we show that the stranded protocol is reproducible and compares well to its non-stranded counterpart [19] and requires little extra hands on time in sample preparation. From our sequencing data we compare the performance of two sequence aligners; Star [20] and Tophat2 [21]. In contrast to the published method [19] we use ribosomal depletion instead of poly adenylation selection to enrich RNA and here we evaluate two ribosomal depletion kits; RiboMinus (Ambion®;) and RiboZero Gold (Epicentre). We then highlight some advantages of stranded libraries by performing a differential expression analysis between strand specific and non-stranded libraries and note how this procedure can be used to probe the annotation of the genome. In conclusion, we turn our attention to high coverage strand specific data to further explore stranded features of the transcriptome; we validate the antisense transcription of the pseudogene PTENP1 which has been shown to be involved in the regulatory network of the expression of the gene PTEN [16], and we report novel transcription in the U2OS cell line.

## Results

### Sample preparation

Apart from the ribosomal depletion step and as described in [19] (and in Methods), all other sample preparation steps; carboxylic acid (CA) purification, cDNA synthesis and library preparation, were carried out on a **M**agnatrix™ 1200 **B**iomagnetic Work**s**tation (MBS) (Nordiag ASA, Oslo, Norway), which is equipped with a 12 tip head suitable for preparing 12 samples in parallel. The stranded protocol differs from the non-stranded protocol in two ways; First, during cDNA synthesis a CA purification step, carried out on the MBS, is introduced after the first strand synthesis after which the second strand synthesis continues as normal except the nucleotide mix includes dUTPs instead of dTTPs. This CA purification step is necessary to remove all the dTTPs prior to second strand synthesis. Second, after library preparation [19, 22], a *second strand digestion* step is added. This step ensures that only the first strand survives the subsequent PCR amplification step and hence the strand information of the libraries. Each of these additional steps add 45-60 minutes to the total preparation time, with about 15-20 min of those being hands on. Additional file 1 shows the main automated steps of sample preparations and highlights the difference between the non-stranded method and the strand specific method.

In total there were 15 libraries prepared, 12 strand specific and 3 non-stranded. All libraries returned a high yield; 78.1 ng/ *μ*l and 110.4 ng/ *μ*l on average for the strand specific and non-stranded libraries respectively. All the libraries had comparable mean fragment length; 259 bp and 245 bp on average for the strand specific and non-stranded libraries, respectively. Additional file 2 shows the concentration and the mean fragment length of each of the 15 libraries.

### Sequencing

All libraries were sequenced on the Illumina HiSeq 2000 generating 100 bp paired end reads. The 15 libraries were divided into 5 groups depending on how they were prepared as shown in Table 1. In Table 1 the Group ID shows the RNA source (A431, U251 or U2OS), the enrichment method (RiboMinus or RiboZero) and the library type (strand specific or non-stranded). Also shown in Table 1 is the average number of raw read pairs generated in the sequencing. The raw sequencing reads are available at the NCBI Sequence Read Archive under accession SRP043027 (SRA, http://www.ncbi.nlm.nih.gov/Traces/sra/).

**Table 1:** **Overview of library groups**

			Raw read pairs
Group No.	Group ID ^∗^	Libraries	(millions)
Group 1	A431_RMSS	1-2	17.0 ±0.1
Group 2	U251_RMSS	3-5	18.7 ±2.7
Group 3	U2OS_RMSS	6-8	16.7 ±0.4
Group 4	U2OS_RMNS	9-11	15.8 ±0.5
Group 5	U2OS_RZSS	12-15	230.2 ±5.8

Grouping of libraries according to RNA enrichment and library type along with the average number, and the standard error, of raw read pairs in each group. [ ^∗^RM = enriched with RiboMinus, RZ = enriched with RiboZero, SS = strand specific, NS = non-stranded. A431, U251 and U2OS denote different cell lines].

### Trimming and mapping

Read alignment was performed by Tophat v2.0.4 [21] and Star v2.3.1o [20] on raw reads and on quality reads, i.e. reads that had been through adapter removal and quality trimming (see Methods). Tophat mapped 59.8% of the raw reads on average compared to 88.2% for Star. For the raw reads the average mapping speed, measured in mapped read pairs per second, was 542 for Tophat compared to 50000 for Star. The percentage of raw reads discarded from analysis by the quality trimming step step was 0.71, 0.40, 0.97, 0.50 and 3.66 for Groups 1, 2, 3, 4 and 5 respectively. Tophat mapped 74.6% of the quality reads on average compared to 94.3% for Star. For the quality reads the average mapping speed, measured in mapped read pairs per second, was 701 for Tophat compared to 64900 for Star. Thus, Star is nearly one hundred times faster than Tophat.

**Figure 1:**
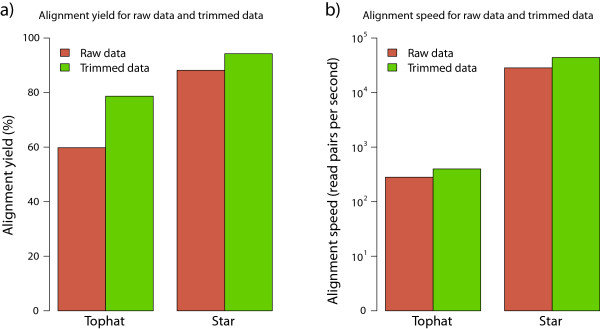
**Alignment yield and mapping speed.** Mapping yield **a)** and mapping speed **b)** for the aligners Tophat and Star, performed both on raw data and quality data. The mapping efficiancy and mapping speed improves using the quality data for both aligners. Star outperforms Tophat both in alignment yield and in mapping speed. Note that the alignment speed is plotted on a log scale.

Graphical representation of these mapping attributes, mapping percentage and mapping speed, for both aligners and a comparison between the handling of raw data and quality data is shown in Figure 1. For these attributes Star outperforms Tophat in all instances. Also, the quality trimming improves the alignment yield, not only in relative terms but in absolute terms as well (see Additional file 3), and the mapping speed. Based on these results all further downstream analysis was based on the quality trimmed data aligned with Star.

### Quality control metrics

The robustness of the protocol and the quality aspects of the data were evaluated using the 15 libraries generated from the human cell lines (libraries 1-15) through different metrics; ribosomal RNA in data, strand specificity, duplication rate, gene body coverage and expression correlation. Details from some of these analyses can be found in Additional file 3.

#### Ribosomal contamination

**Figure 2:**
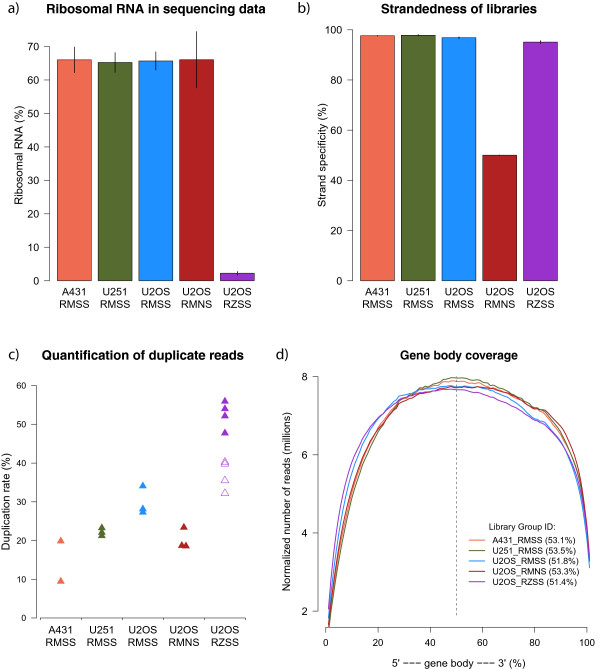
**Quality control metrics for human cell line libraries.** **a)** rRNA content in libraries treated with RiboZero is 2.24% on average while rRNA content in libraries treated with RiboMinus is 65.7% on average. Error bars denote standard error. **b)** The strand specificty of strand specific libraries is 96.6% on average; the libraries treated with RiboMinus have slightly higher strand specificity than the libraries treated with RiboZero. The unstranded libraries have strand specificity of 50.0%. **c)** The duplication rate varies between the libraries. The higher duplication rate of the libraries treated with RiboZero compared to the libraries treated with RiboMinus can partly be explained by the much higher sequencing depth of those libraries. The hollow triangles represent the duplication rate of the downsampled data (see text for details). **d)** All libraries show even gene coverage. The percentages in parenthesis is the percentage of reads that map closer to the 3’ end than to the 5’ end. [RM = RiboMinus, RZ = RiboZero, SS = strand specific, NS = non-stranded].

To evaluate the efficiency of the ribosomal depletion, the rRNA reads in each library were quantified. On average the libraries treated with RiboMinus contained 65.7% rRNA compared to only 2.24% for the libraries treated with RiboZero. Figure 2a shows the average percentage of rRNA reads in each library group.

#### Strandedness of libraries

Figure 2b shows the average strand specificity of each library group. Here, strand specificity means the percentage of times the read matches the annotation correctly according to how the library was made. For dUTP libraries the first read in a read pair must be reversed so it matches the annotation while the second read matches the annotation directly. For the strand specific libraries treated with RiboMinus (library groups 1-3) the average strand specificity is 97.4% while for the strand specific libraries treated with RiboZero (library group 5) the average strand specificity is 95.1%. This difference was found to be statistically significant (Student’s t-test, p < 0.05). The average strand specificty for the non-stranded libraries (library group 4) is 50.0% as expected.

In general, these results show that the protocol works and in particular that the CA purification step is successful in removing dTNPs after the first strand synthesis.

#### Duplication quantification

Figure 2c shows the average percentage of duplicates identified for each library group. There is some variation in duplication frequency in the libraries especially between library groups. The libraries treated with RiboZero show higher duplication rate, 52.5% on average, than the libraries treated with RiboMinus, 22.4% on average. This difference was thought to be related to the difference in sequencing depth. To verify that, the RiboZero data was downsampled to 15 million reads and the duplication rate quantified again. The duplication rate decreased from 52.5% to 37.0% when using the downsampled data and thus the high duplication rate can only be explained partly by the high sequencing depth. The duplication rate for this downsampled data is shown as hollow symbols in Figure 2c.

Due to the risk of inaccurately identifying reads originating from highly expressed genes as duplicates, the duplicate reads were not removed prior to differential expression analysis.

#### Gene coverage and read distribution

Figure 2d shows the read coverage, normalized for the different read depths, and averaged over each library group. No discernable difference can be seen between the libraries and they all show even coverage across the gene body. Additional file 4 shows, for each group, how the reads are distributed to exons, introns and intergenic regions. Analysis of variance (ANOVA) revealed no significant difference in the read distribution between the groups.

#### Expression correlation

To further assess the robustness of the libraries the correlation of expression values between replicates was quantified. The mean Pearson correlation of the 16 possible correlations within replicates was *R*^2^ = 0.96. Correlation plots and the Pearson correlation value for each correlation is shown in Additional file 5.

### Differential expression - strand specific vs. non-stranded data

The only difference between libraries in group 3 and group 4 is that the libraries in group 3 are strand specific, generated using the current approach, while the libraries in group 4 are non-stranded, generated using the approach in [19]. In order to explore the differences between these library types a differential expression (DE) analysis was carried out beween these groups; first by downsampling each library so that they contain equal amount of reads, then by acquiring read counts per gene using htseq-count [23] and finally using these read counts as input for the DE analysis using DESeq v1.10.1 [24].

**Figure 3:**
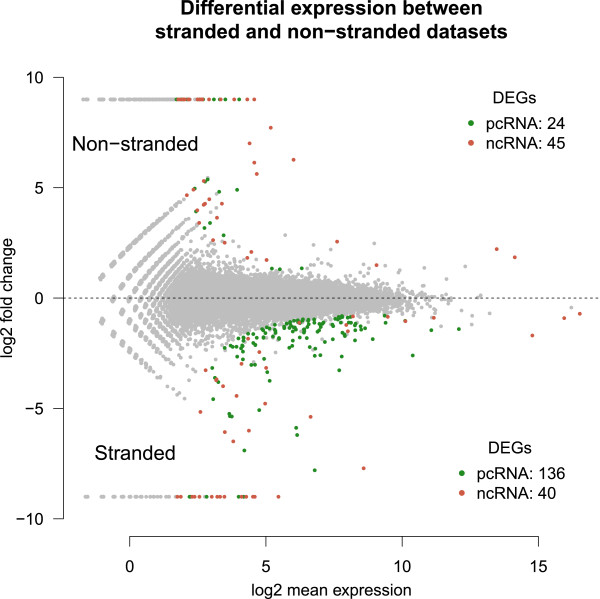
**Differential expression profile when comparing the strand specific libraries to the non-stranded libraries.** All green dots are protein coding genes found to be signficantly differentially expressed and all red dots are non coding genes found to be signficantly differentially expressed. pcRNA: protein coding RNA, ncRNA: non-coding RNA.

Of the 62893 annotated genes (protein coding and non-coding) 41065 do get assigned low or no expression in all of the six libraries. Of the remaining 21828 genes 245 are found to be significantly differentially expressed genes, hereafter referred to as DEGs. Of these 245 DEGs 69 have higher expression in the non-stranded libraries while 176 DEGs have higher expression in the stranded libraries. Intriguingly the division of DEGs into protein coding genes and non coding genes is different depending on whether the DEGs have a higher expression in the non-stranded data or in the stranded data. So, for the 69 DEGs which show higher expression in the non-stranded data 24 are protein coding and 45 are non-coding while for the 176 DEGs which show higher expression in the stranded data 136 are protein coding and 40 are non coding. This expression profile is shown in Figure 3.

To find out why these DEGs arise, coverage plots for a selection of the DEGs with the lowest p-values, were analysed and compared to the annotation used for counting by htseq-count. All DEGs investigated that have a higher expression in the stranded data compared to the non-stranded data have overlapping annotation which results in many reads mapping to those genes being labeled as ambiguous for the non-stranded data and hence resulting in low expression. Explanation for DEGs with higher expression in the non-stranded data compared to the stranded data is not as straightforward but scrutiny revealed three dominant reasons for these DEGs; i) the DEGs have overlapping features that are unnannotated, ii) the DEGs are annotated in the wrong direction or iii) the DEGs have antisense intronic transcripts that get wrongly assigned to them. Additional file 6 shows coverage plots of selected DEGs along with their annotation and explanations for why these DEGs arise in this comparison and Additional file 7 lists all the genes found to be significantly differentially expressed in this differential expression analysis.

This analysis also demonstrates how essential it is to have strand specific libraries for compact genomes with high abundances of overlapping genes since without strand specificity a large proportion of the genes would be labeled as ambiguous.

### Transcriptome assembly - strand specific vs. non-stranded data

For each library in group 3, 4 and 5 two transcript assemblies were made using Cufflinks [25]. The first, termed raw assembly, used all mapped reads while the other, termed novel assembly, used only those reads that did not map to the ensembl reference annotation (version GRCh37.72). Then the assemblies within each group were merged together using Cuffmerge [25]. In addition, library 5 was assembled again without supplying Cufflinks with the information that it was strand specific thus generating a pseudo non-stranded assembly.

From these assemblies it was found that strand specific data generates fewer transcripts compared to non-stranded data and the average transcript length is usually shorter for the strand specific data as compared to the non-stranded data. The same holds true when comparing the assemblies between library group 5 treated as strand specific to library group 5 treated as non-stranded. An overview of the assembly results is shown in Additional file 8.

### Antisense transcription of the pseudogene PTENP1

The antisense transcription of the pseudogene PTENP1 has previously been reported and suggested to play a role in the regulation of the gene PTEN [16]. From the high coverage data of the U2OS cell line (Library Group 5) this antisense transcription was verified and further evaluated.

**Figure 4:**
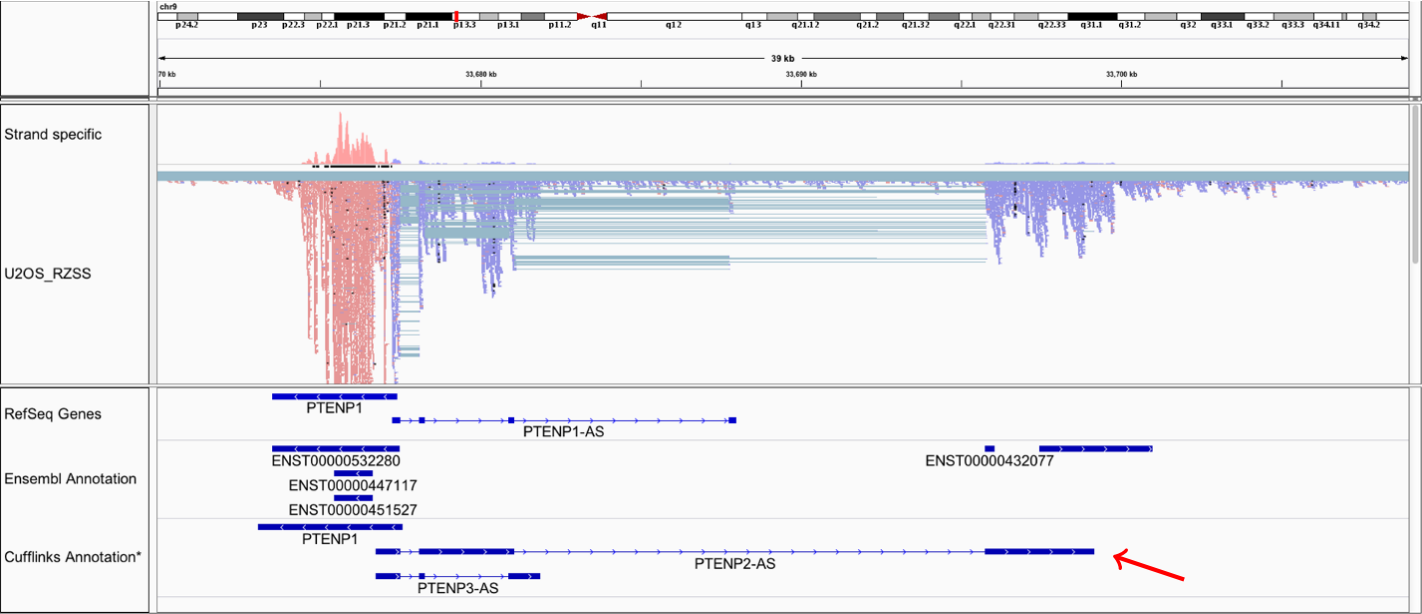
**Antisense RNA of PTENP1.** Antisense transcription of the pseudogene PTENP1. The RefSeq annotation includes only one isoform (top annotation track) while the Ensembl annotation does not have this transcript annotated. Our data, based on Cufflinks assembly, suggest two alternative isoforms for this transcript labeled PTENP1-AS2 and PTENP1-AS3. The PTENP1-AS2 isoform includes a novel exon, higlighted by a red arrow, which overlaps ensembl annotation of other genes. It is suggested here that this ensembl annotation is wrong and that these genes are part of the PTENP1-AS gene. *The Cufflinks assembly shown here has been cleaned up a bit. For the direct output delivered by Cufflinks see Additional file 12.

The coverage plot in Figure 4 shows clear antisense expression of the PTENP1 pseudogene. The RefSeq database has this antisense transcript annotated as a gene with one isoform containing four exons as shown in the uppermost annotation track in Figure 4 (PTENP1-AS). The Ensembl database, however, does not have this antisense transcript annotated but it does have two genes annotated further downstream as shown in the Ensembl annotation track in Figure 4. The new annotation presented here, based on the raw assembly results from library group 5, is shown at the bottom annotation track in Figure 4 which suggests two new isoforms of the antisense gene PTENP1 one of which includes a new exon (PTENP1-AS2). This new exon overlaps the two annotated genes from the Ensembl annotation indicating that they may be, not seperate genes, but part of the PTENP1 asRNA.

Using an annotation which included the RefSeq isoform, PTENP1-AS, and our two new isoforms, PTENP1-AS2 and PTENP1-AS3, the isoform expression levels were evaluated with Cufflinks. PTENP2-AS is expressed with an average FPKM value of 0.64 but the other isoforms, PTENP1-AS and PTENP3-AS, showed no expression according to Cufflinks.

### Novel expression in U2OS

Identification of novel genes was attempted by using the novel assembly from library group 5. By counting the mapped reads towards this novel assembly the most highly expressed genes were investigated. Many of the assembled transcripts were evidently intron transcripts and others matched the current annotation, at least partly. Other transcripts were potentially novel genes.

**Figure 5:**
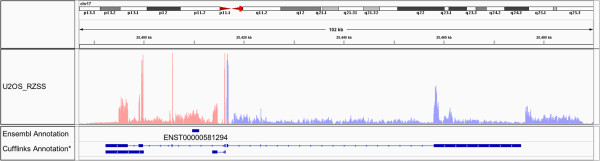
**Novel expression in U2OS.** Novel, cell specific, expression on chromosome 17. The coverage plot shows transcriptional activity, color coded to reflect the strand of origin. In the Ensembl database this region has one annotated pseudogene. The data here indicate high transcriptional activity from both strands. Shown at the bottom is the annotation as suggested by Cufflinks. *The Cufflinks assembly shown here has been cleaned up a bit. For the direct output delivered by Cufflinks see Additional file 13.

One interesting example are two overlapping novel genes on chromosome 17: 25380000-25500000. Figure 5 shows this region along with the annotations from Ensembl and the prediciton by Cufflinks. Currently the only known annotation in this region is the pseudogene TUFMP1 (ENST00000581294) but the data shown here clearly indicates more transcriptional activity, originating from both strands. The open reading frame of these novel transcripts indicates that they are non-coding. Comparing the transcription of the locus between the three cell lines shows that this transcription is exclusive to the U2OS cell line (see Additional file 9).

Two other interesting findings can be found in the Additional files; a U2OS cell specific transcription on chromosome 6, possibly a pseudogene, is shown in Additional file 10, and ubiquitous transcription of chromosome 14, which is currently annotated as two genes but our data suggests it is two exons of one gene, is shown in Additional file 11.

## Discussion

### Sample preparation

We have modified an existing non-stranded automated RNA library protocol into a protocol that generates strand specific libraries by using the Illumina TruSeq kit in combination with other reagents. This may be useful for other researchers who use the Illumina TruSeq kit and want to make strand specific libraries. However, Illumina has now released a new kit; TruSeq Stranded Total RNA Sample Prep Kit (# RS-122-2201), which combines RiboZero rRNA depletion with a strand specific method similar to the dUTP method. It should be possible to fully automate that protocol on the MBS for generating 12 samples in parallel or on the Agilent Bravo for a higher throughput of 96 samples in parallel.

### Trimming and mapping

We showed that quality trimming and adapter removal improves the alignment yield and mapping speed, both for Tophat and Star. We set the quality threshold to 20 on the Phred scale and then removed any pairs that contained reads shorter than 20 bp after trimming. By setting the parameters differently this improvement might be different. For example by increasing the quality threshold it is likely that the mapping percentage will increase but that will not neccessarily improve the mapping in absolute terms since many more reads would be discarded prior to mapping. Due to trimming, some of the quality reads are shorter than the raw reads which could explain the improvement in mapping speed of the quality data over the raw data.

We also showed that Star outperforms Tophat in alignment yield and mapping speed. This is in accordance with previous reports on alignment yield [20, 26] and mapping speed [20]. There are other features where these two aligners differ which are not investigated here but a recent and thorough comparison of various spliced aligners can be found in [26].

### Quality control metrics

We showed that the RiboZero Gold kit far outperforms the RiboMinus kit in removing ribosomal molecules from RNA samples. It should be noted that the RiboMinus kit is now no longer available for purchase and has been replaced by RiboMinus v2. We show a 2.2% rRNA contamination in the RiboZero treated samples which is better efficiency than previously showed in a comparison study [27].

#### Duplication quantification

The markedly higher amount of duplicate reads in the RiboZero libraries compared to the RiboMinus libraries can partly be explained by the much higher sequencing depth of those samples. Still, all the libraries show a considerably high duplication rate. We believe that this can be explained by too many PCR cycles in the libary preparation but in the protocol the number of PCR cycles was fixed at 15. We have now altered the protocol to include a qPCR analysis step to measure the Ct value (concentration threshold in qPCR) which better determines the amount of PCR cycles needed for each library during preparation.

### Differential expression - strand specific vs. non-stranded data

We compare our current, strand specific, approach to a previously, non-stranded, approach [19] and show that genes get determined as differentially expressed when comparing strand specific data to non-stranded data, from otherwise identical samples. Out of 21828 expressed genes we identify 245 of them, or around 1.1%, to be differentially expressed.

We further showed that these genes arise in a systematic manner in such a way that overlapping genes that are annotated get underrepresented in the non-stranded data and genes that have a faulty annotation can get overrepresented in the non-stranded data. Also, genes in the non-stranded data can get overrepresented due to intronic transcripts from the opposing strand.

This highlights the importance of having strand specific libraries in order to be better able to make correct assumptions from the data. It also emphasizes the need for accurate and verified annotation which, currently, even for human is erronous and incomplete. By comparing stranded and non-stranded data it is possible to probe the areas of the annotation that need attention. This also shows, in the absence of strand specific data, that researchers need to be extra attentive when interpreting results from overlapping loci in the genome.

It should be noted that this method may not be sensitive enough to pick up all antisense behavior, for example if the overlap between the two strands is only a small proportion of the transcribed genes, and evidently more than 1.1% of the genome features antisense transcription.

### Transcriptome assembly

From the transcriptome assembly we show that the strand specific data produces fewer transcripts than the non-stranded data. Also, strand specific data usually produces shorter transcripts. This may indicate that assemblies from non-stranded data generates more false positives than does strand specific data.

It must be stressed though that Cufflinks is far from perfect and tends to generate many questionable transcripts as can be seen from the transcripts we removed (see Additional files 12 and 13) and the many transcripts assembled from library group 5 (see Additional file 8). Cufflinks also has many different parameters that can be tweaked which can effect the results signficiantly. Assembling transcripts is a difficult task and is not the major focus of the current study. Nevertheless, Cufflinks proved useful as a guide for the asemblies we present and to highlight one of the many differences between strand specific and non-stranded data.

## Conclusions

The Illumina TruSeq library preparation kit can, with modifications, be used to make strand specific libraries. The RiboZero kit is excellent in removing rRNA molecules from total RNA and the Star aligner is ideal for big datasets and/or when time is a factor in the analysis and we show that quality trimming can improve mapping efficiency. There is a good selection of freely available bioinformatical tools for RNA sequencing analysis many of which have an option to indicate whether the data is strand specific or non-stranded. Thus there is, computationally, not much difference in analysing strand specific data compared to analysing non-stranded data. Furthermore, none of these tools are specialized for handling strand specific data nor are any of them at a disadvantage when applied to strand specific data. However, data from strand specific libraries is more reliable than data from unstranded libraries and can correctly evaluate the expression of asRNA and other overlapping genes as well as the direction of intronic reads. The annotation of the human genome is comparatively thorough and correct but still in need for verification, correction and improvement, all of which can be achieved with strand specific RNA sequencing.

## Methods

For many of the different analysis we use various freely available command line tools. The command line for selected tools is given in Additional file 14.

### Ethics approval statement

The study uses three well established cancer cell lines available from certified providers; U251 (Professor Bengt Westermark, Uppsala University), A431 (DSMZ) and U2OS (ATCC-LGC). The cell lines were cultured as suggested by the providers and as previously described [28].

### Experimental design

For the evaluation of the protocol 15 libraries were used whose attributes are showed in Table 2. Libraries 1-2, 3-5 and 6-15 were made from RNA from the cell lines A431, U251 and U2OS respectively. The RNA for libraries 1-11 was enriched with RiboMinus (Ambion®;) while RNA for libraries 12-15 was enriched with RiboZero (Epicentre). Libraries 1-8 and 12-15 were prepared in a strand specific manner while libraries 9-11 were prepared in a non-stranded manner.

**Figure 6:**
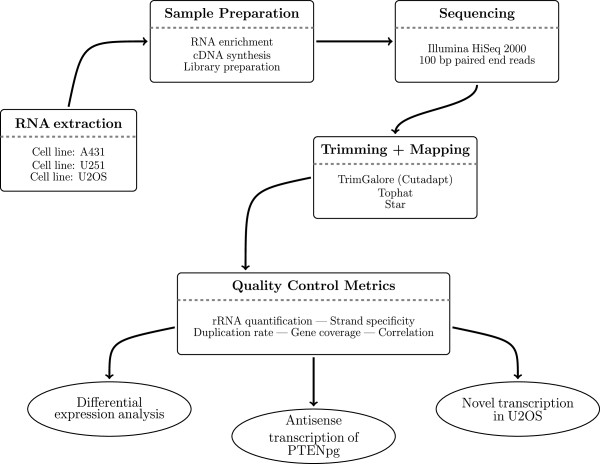
**Flowchart giving an overview of the experimental design.**

From these libraries the data was explored in distinct steps as outlined in Figure 6. Briefly, all the libraries were sequenced on Illumina HiSeq 2000 generating 100 bp paired end reads. The reads were then quality trimmed before being mapped to the genome. After mapping, the data from the human cell lines were analyzed through various quality control metrics before being further explored by differential expression analsysis, verifying antisense transcription of PTENP1 and identifying novel transcription in the U2OS cell line.

**Table 2:** **Libraries used and their attributes**

Library no.	Library ID	RNA source	RNA enrichment	Library type
Library 1	A431_RMSS_R1	A431 cell line	RiboMinus	Strand specific
Library 2	A431_RMSS_R2	A431 cell line	RiboMinus	Strand specific
Library 3	U251_RMSS_R1	U251 cell line	RiboMinus	Strand specific
Library 4	U251_RMSS_R2	U251 cell line	RiboMinus	Strand specific
Library 5	U251_RMSS_R3	U251 cell line	RiboMinus	Strand specific
Library 6	U2OS_RMSS_R1	U2OS cell line	RiboMinus	Strand specific
Library 7	U2OS_RMSS_R2	U2OS cell line	RiboMinus	Strand specific
Library 8	U2OS_RMSS_R3	U2OS cell line	RiboMinus	Strand specific
Library 9	U2OS_RMNS_R1	U2OS cell line	RiboMinus	Non-strand specific
Library 10	U2OS_RMNS_R2	U2OS cell line	RiboMinus	Non-strand specific
Library 11	U2OS_RMNS_R3	U2OS cell line	RiboMinus	Non-strand specific
Library 12	U2OS_RZSS_R1	U2OS cell line	RiboZero	Strand specific
Library 13	U2OS_RZSS_R2	U2OS cell line	RiboZero	Strand specific
Library 14	U2OS_RZSS_R3	U2OS cell line	RiboZero	Strand specific
Library 15	U2OS_RZSS_R4	U2OS cell line	RiboZero	Strand specific

### Sample preparation

The cell lines A431 (skin carcinoma), U251 (brain glioblastoma) and U2OS (bone osteosarcoma) were cultivated, grown and harvested as described earlier [28]. The RNA was extracted using the RNeasy Mini Kit (Qiagen) according to the manufacturer’s protocol. Quality of RNA samples was assessed using BioAnalyzer 2100 and a Qubit quantification fluorometer. All RNA samples used were of high quality (RIN > 9) and with a concentration between 400 ng/ *μ*l and 800 ng/ *μ*l. Libraries were constructed as explained above and shown in Table 2. The amount of input material for all libraries was 2 *μ*g of total RNA.

#### The library preparation protocol

The automation was set up on a Magnatrix TM 1200 Biomagnetic Workstation (NorDiag ASA, Oslo, Norway) which is equipped with a 12 tip head and is capable of running custom made scripts. The robot features an in tip magnet processing and a Peltier unit (4-95 C) where the reactions were performed.

Our automatic strand specific RNA sequencing library preparation protocol is an adaptation of the dUTP second strand marking protocol utilizing the automation of the Illumina TruSeq protocol along with purification steps using CA beads. The details of the dUTP protocol have been described previously in [7, 18] and the automation of the Illumina TruSeq protocol along with the carboxyl acid (CA) purification steps are described in [19, 22].

Briefly, the steps of our strand specific protocol are: first strand cDNA synthesis; CA purification; second strand synthesized using dUTPs instead of dTTPs; end reapair, A-tailing and adaptor ligation; second strand digestion with UNG; PCR amplification; and CA purification. A flow diagram, highlighting the main differences between the non-stranded and strand specific protocols, is shown in Additional file 1.

#### Clustering and sequencing

The clustering was performed on a cBot cluster generation system using a HiSeq paired-end read cluster generation kit according to the manufacturer’s instructions. All libraries were sequenced on an Illumina HiSeq 2000 as paired-end reads to 100 bp. Base conversion was done using Illumina’s OLB v1.9.

### Trimming and mapping

The raw sequencing data were processed through a quality trimming process before being mapped to the genome. The reads were mapped to the GRCh37.72 primary assembly of the human genome (ensembl.org) using both Tophat2 v2.0.4 [21], and STAR v2.3.1o [20] and their results and performance compared (see Results). To evaluate the effects of trimming on mapping the reads were mapped both before and after trimming. When reads mapped to multiple locations only the primary hits were retained.

For the quality and adapter removal the utility program Trim Galore! [29] was used. Trim Galore! is a wrapper script that makes use of the trimming tool cutadapt [30]. Possible adapter sequences, based on the Illumina TruSeq Adapter index sequences, were removed from the reads. The reads were then quality trimmed, with a quality threshold of 20 on the Phred scale, and if either read from a pair was shorter than 20 bp after trimming that pair was removed from the analysis.

### Quality Control Metrics

Selected scripts from the quality control package RSeQC [31] were used to assess quality metrics from the data; split_bam.py for ribosomal quantification, infer_experiment.py for strand specificity and geneBody_coverage.py for gene coverage. The duplication rate was quantified using MarkDuplicates from Picard [32].

To count read expression the program htseq-count [23] was used. It uses a gene transfer format (GTF) annotation file, downloaded from the Ensembl database (version GRCh37.72), as a reference and and assigns reads to a feature (a gene), or labels them as matching to no feature or as ambiguous if it matches more than one feature and it cannot determine which one it is. Genes that have fewer number of reads than the total number of assigned reads divided by one million were labeled as lowly expressed. If a gene had zero or low expression in both datasets being compared, such as in correlation and differential expression analysis, that gene was omitted from that comparison. This filtering step was included to try and reduce false positives in the comparisons [33]

### Differential expression - strand specific vs. non-stranded data

For the differential expression (DE) analysis the filtered output from htseq-count was used as an input for the R package DESeq [24]. Prior to counting, all samples in the DE analysis were downsampled to 4.5 million sequences to ensure equal amount of reads in all libraries being compared. The downsampling was carried out using DownsampleSam from Picard tools [32]. All genes with a p-value of 0.05 or below after Benjamini-Hochberg ajdustment were labeled as differentially expressed genes (DEGs). Using the annotation from the Ensembl database the DEGs were categorized into protein coding genes and non-coding genes. The IGV genome browser [34] was used for visualization of selected DEGs.

### Transcriptome assembly - strand specific vs. non-stranded data

The assembly was carried out using Cufflinks [25] to generate two kinds of assemblies; *raw assembly* and *novel assembly*. For the raw assembly all mapped reads were used and no reference annotation was used to guide the assembly. All parameters were kept at default values except for the stranded libraries the parameter ‘–library-type’ was set to ‘fr-firststrand’. After the assembly the assemblies within each group were merged using Cuffmerge.

For the novel assembly the mapped reads were split, using split_bam.py from RSeQC [31], into two bam files; the reads that matched the annotations and the ones that did not match the annotation. Then only those reads that did not match the annotation were used as input for Cufflinks. To further ensure the assembly of novel transcripts the current annotation was masked from the assembly using the ‘-M’ option.

### Antisense transcription of PTENP1

The IGV genome browser was used to visualize the coverage of the PTENP1 locus along with annotations from RefSeq and Ensembl. From the raw assembly from group 5 new annotation for the PTENP1 asRNA was constucted. This new annotation was then used to evaluate its isoform expression.

### Novel annotation in U2OS

Htseq-count was used to determine the expression of the novel assembly from library group 5. Then the coverage plots of the highest expressed ‘novel’ genes were investigated using the IGV browser. Many of the alleged ‘novel’ genes turned out to be intronic transcripts wrongly assembled as exons and others overlapped current annotation. Manual searching, however, revealed potentially novel expression with a selected few represented in this study.

## Availability of supporting data

All the raw sequencing reads have been submitted to the NCBI Sequence Read Archive and are available under accession SRP043027 (SRA, http://www.ncbi.nlm.nih.gov/Traces/sra/).

## Electronic supplementary material

Additional file 1: **Figure S1.** Flow diagram of the library protocol, highlighting the difference between the non-stranded and strand specific approach. (PDF 68 KB)

Additional file 2: **Table S1.** Overview of the concentration and average fragment length of each library after sample preparation. (XLSX 50 KB)

Additional file 3: **Table S2.** Details of the data processing of each library. (XLSX 51 KB)

Additional file 4: **Figure S2.** Distribution of reads into exonic, intronic and intergenic regions for each library group. (PDF 53 KB)

Additional file 5: **Figure S3.** Expression correlation between all libraries within the same group. (PDF 671 KB)

Additional file 6: **Figure S4:** **A-H.** Coverage plots along with explanations for some of the differentially expressed genes found when comparing the expression of non-stranded data to strand specific data. (PDF 498 KB)

Additional file 7: **Table S3.** List of all differentially expressed genes found in the DE analaysis between non-stranded and strand specific data. (XLSX 72 KB)

Additional file 8: **Table S4.** Overview of the Cufflinks assembly results. A comparison between non-stranded and strand specific data. (XLSX 51 KB)

Additional file 9: **Figure S5.** Coverage plot showing that the novel transcription shown in Figure 5 in the main text is exclusive to the U2OS cell line. (PDF 91 KB)

Additional file 10: **Figure S6.** Another novel transcription exclusive to the U2OS cell line. (PDF 80 KB)

Additional file 11: **Figure S7.** Coverage plot indicating that two exons annotated as two genes may actually be two exons from the same gene. (PDF 91 KB)

Additional file 12: **Figure S8.** The raw transcript assembly around the PTENP1 locus highlighting the manual changes made for the proposed assembly. (PDF 133 KB)

Additional file 13: **Figure S9.** The raw transcript assembly around the locus of the novel gene in Figure 5 highlighting the manual changes made for the proposed assembly. (PDF 92 KB)

Additional file 14: **Supplementary file S1.** File listing some of the command lines used for the analysis. (PDF 87 KB)
