# Supplementary material for: Analysis of stranded information using an automated procedure for strand specific RNA sequencing
Source: BMC Genomics. 2014 Jul 28;15(1):631. doi: 10.1186/1471-2164-15-631 (PMC4247151; doi:10.1186/1471-2164-15-631)
Supplement: Supplementary file 1 — Additional file 1: Figure S1. Flow diagram of the library protocol, highlighting the difference between the non-stranded and strand specific approach. (PDF 68 KB) [file 12864_2014_6674_MOESM1_ESM.pdf]

Analysis of stranded information using an automated procedure for strand specific RNA sequencing

Additional file 1

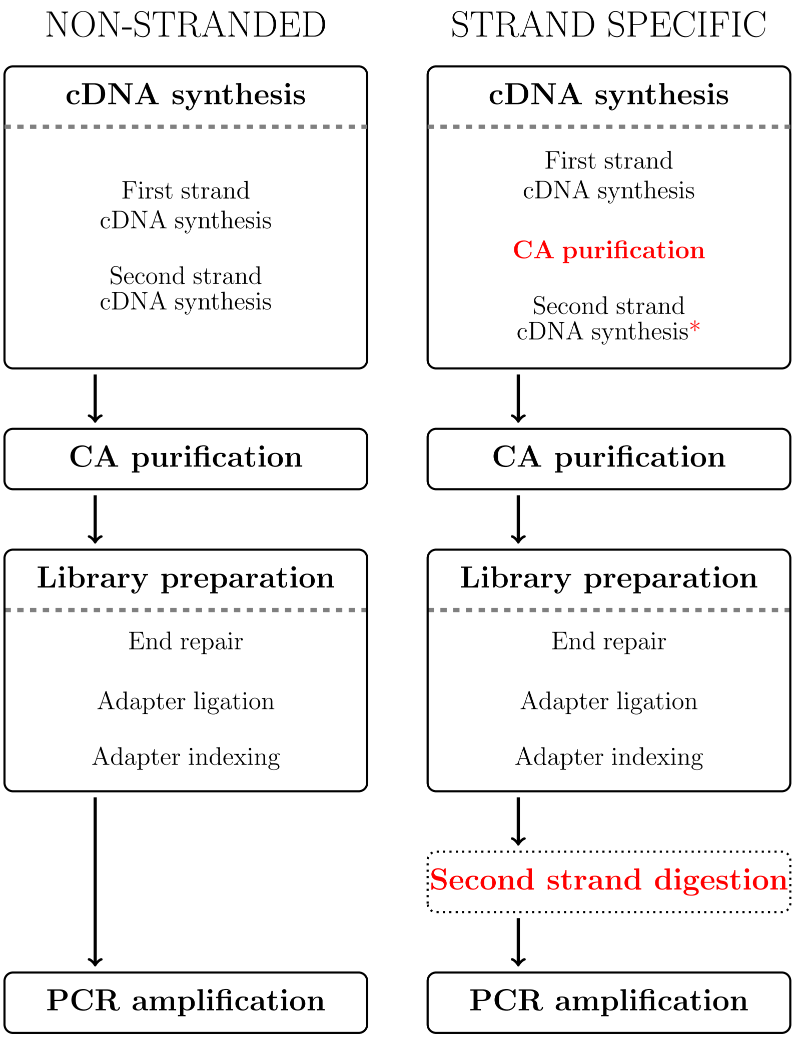

Figure S1: Overview and comparison of the non-stranded and strand specific protocols. The automation of the non-stranded protocol can be found in [19, 23]. Only two steps are needed to modulate the non-stranded method into the strand specific method; First, a CA purification step is introduced between the first and second strand synthesis step. This is done to remove all dNTPs prior to the second strand synthesis which is done using dUTPs instead of dTTPs. Second, after library preparation the second strand is digested using Uracil-N-Glycosylase (UNG) which ensures that only the first strand survives the subsequent PCR amplification step.
