# Supplementary material for: Analysis of stranded information using an automated procedure for strand specific RNA sequencing
Source: BMC Genomics. 2014 Jul 28;15(1):631. doi: 10.1186/1471-2164-15-631 (PMC4247151; doi:10.1186/1471-2164-15-631)
Supplement: Supplementary file 4 — Additional file 4: Figure S2. Distribution of reads into exonic, intronic and intergenic regions for each library group. (PDF 53 KB) [file 12864_2014_6674_MOESM4_ESM.pdf]

Analysis of stranded information using an automated procedure for strand specific RNA sequencing

Additional file 4

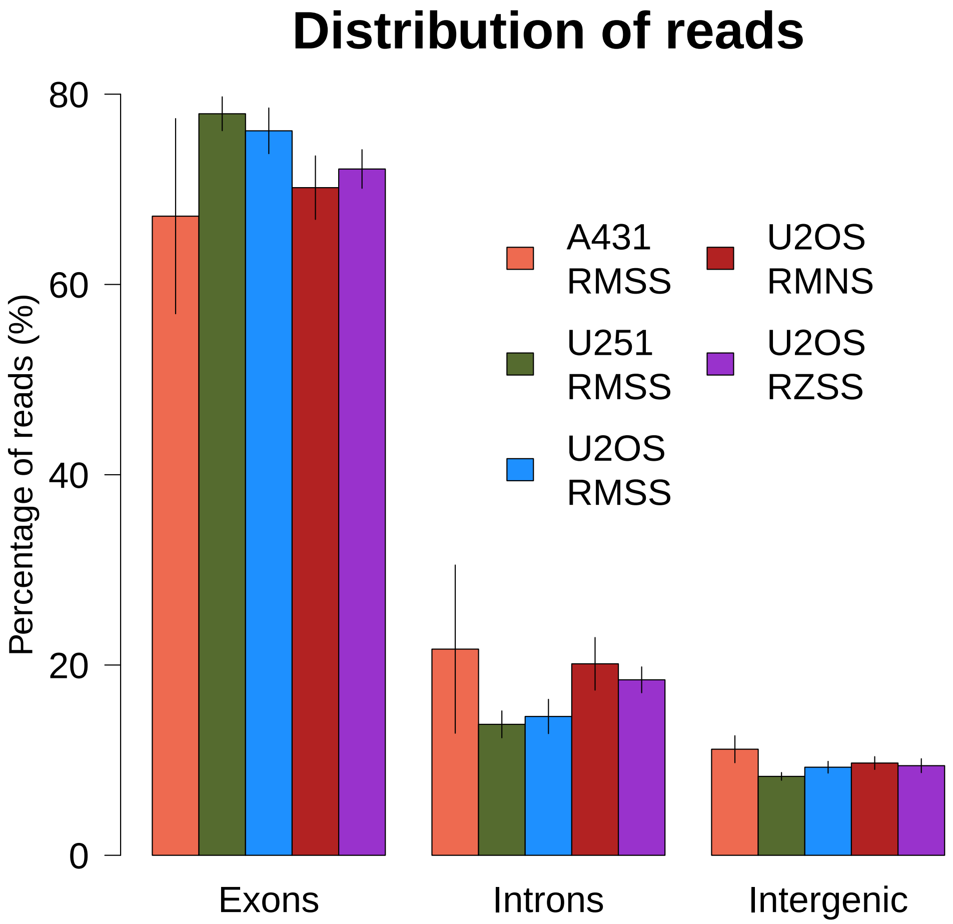

Figure S2: Distribution of mapped reads into exonic, intronin and intergenic regions. Shown are the averages for each of the five library groups and error bars denote standerd error. The distribution is similar between groups and ANOVA rvealed no significant difference between them. Noteworthy are the large error bars for the A431_RMSS group. That group only contained two libraries, one of which showed unusuall high mapping to intronic regions.
