# Supplementary material for: Analysis of stranded information using an automated procedure for strand specific RNA sequencing
Source: BMC Genomics. 2014 Jul 28;15(1):631. doi: 10.1186/1471-2164-15-631 (PMC4247151; doi:10.1186/1471-2164-15-631)
Supplement: Supplementary file 5 — Additional file 5: Figure S3. Expression correlation between all libraries within the same group. (PDF 671 KB) [file 12864_2014_6674_MOESM5_ESM.pdf]

Analysis of stranded information using an automated procedure for strand specific RNA sequencing

Additional file 5

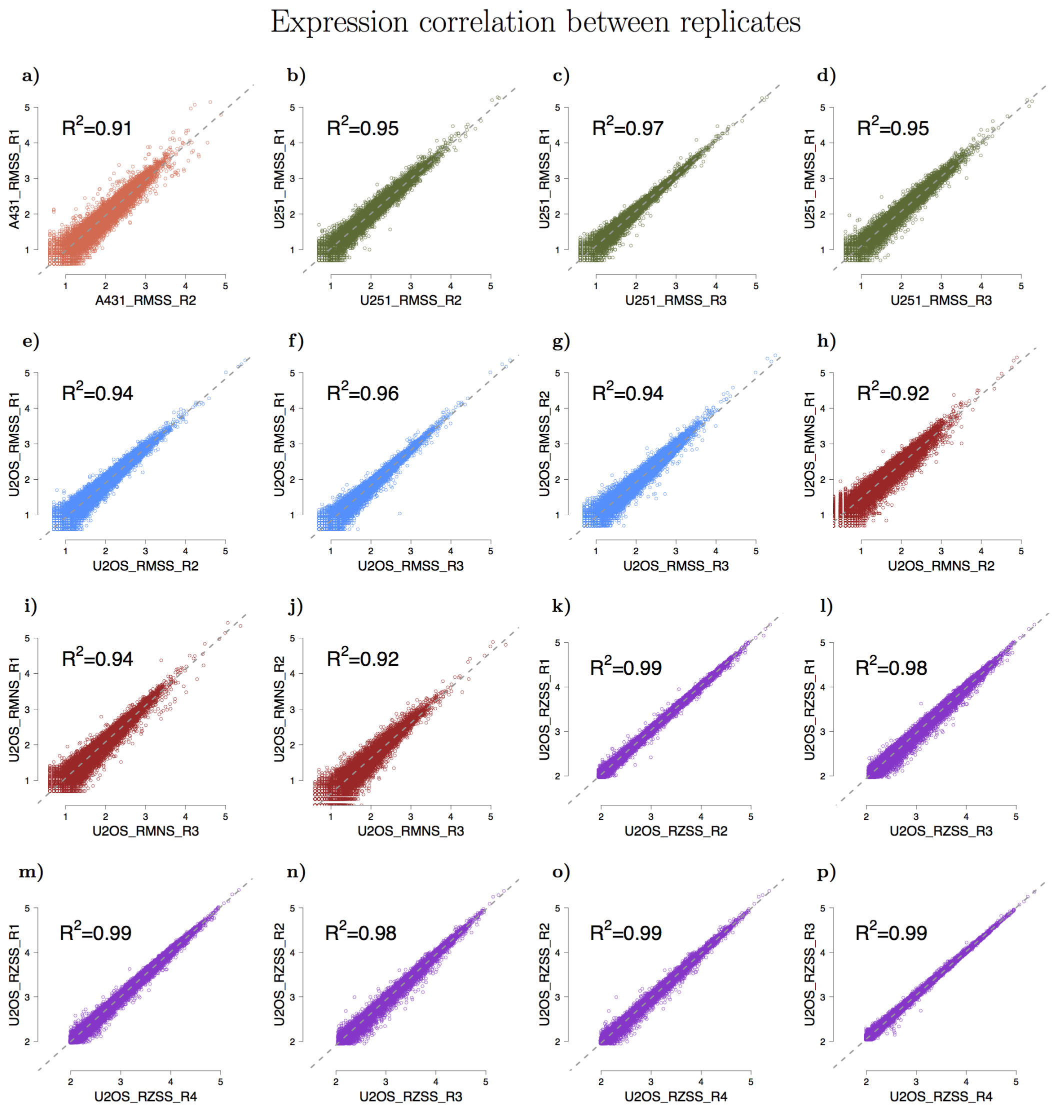

Figure S3: Correlation of expression values between replicates. All 16 correlations between replicates, but within groups, have an average Pearson correlation coefficient of 0.96. Values on all axes are expression counts on a $\log_{10}$ scale, ie. $1 = 10^1$, $2 = 10^2$, etc. The plots are color coded to indicate within which group the correlation takes place; a) A431_RMSS, b)-d) U251_RMSS, e)-g) U2OS_RMSS, h)-j) U2OS_RMNS and k)-p) U2OS_RZSS.
