# Supplementary material for: Analysis of stranded information using an automated procedure for strand specific RNA sequencing
Source: BMC Genomics. 2014 Jul 28;15(1):631. doi: 10.1186/1471-2164-15-631 (PMC4247151; doi:10.1186/1471-2164-15-631)
Supplement: Supplementary file 6 — Additional file 6: Figure S4: A-H. Coverage plots along with explanations for some of the differentially expressed genes found when comparing the expression of non-stranded data to strand specific data. (PDF 498 KB) [file 12864_2014_6674_MOESM6_ESM.pdf]

Analysis of stranded information using an automated procedure for strand specific RNA sequencing

Additional file 6

This file comprises Figure S4:A-H which show and explain selected results from the differential expression analysis between non-stranded and strand specific data. All the figures are generated from the IGV genome browser and below is a small explanatory figure describing the basic features of the figures as well as a small overview of the contents of this file.

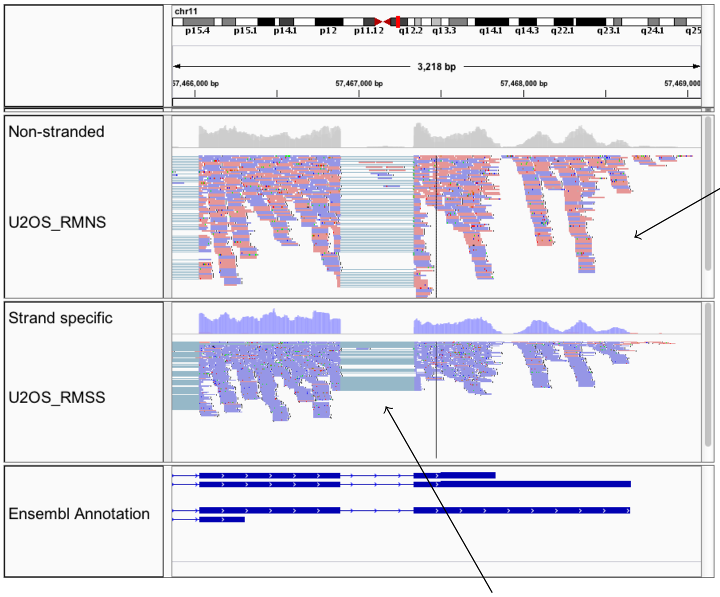

Reads are color coded depending on whether they are read 1 or read 2 of the paired end reads.

For the strand specific data this indicates correctly from which strand the read comes from. No such information is available from the non-stranded data.

When reads are spliced it is shown with gray-green lines.

Contents:

A. Overlapping annotation, Chr 11 2
B. Overlapping annotation, Chr 18 3
C. Unannotated overlapping feature, Chr 17 4
D. Unannotated overlapping feature, Chr 8 5
E. Annotation in the wrong direction, Chr 10 6
F. Annotation in the wrong direction, Chr X 7
G. Antisense intronin transcripts, Chr 7 8
H. Antisense intronin transcripts, Chr 11 9

A Overlapping annotation
Chr11:66240000-66340000

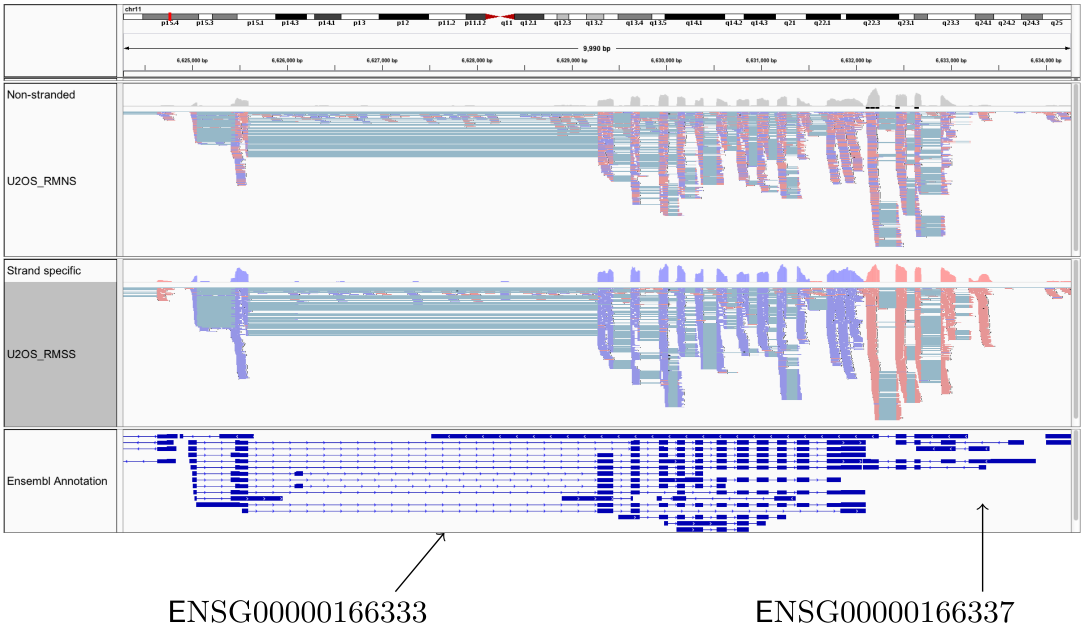

Protein coding gene found to be significantly higher expressed in the strand specific data compared to the non-stranded data. This gene, ENSG00000166333, has 17 annotated isoforms, of which 10 are protein coding, shown at the annotation track at the bottom of the figure. The annotation also shows an overlapping protein coding gene, ENSG00000166337, transcribed in the opposite direction. Note, especially, the long overlapping exon at the top of the annotation track. All reads that map to any overlapping exons of these two genes are labelled as ambiguous for the non-stranded data (top coverage track) hence its low expression as determined by htseq-count. For the stranded data, however, htseq-count has no problem of assigning the reads to the overlapping genes. This scenario appears to be dominant in explaining why genes get labelled as being significantly higher expressed in the stranded data compared to the non-stranded data.

B Overlapping annotation
Chr18:29990000-29995000

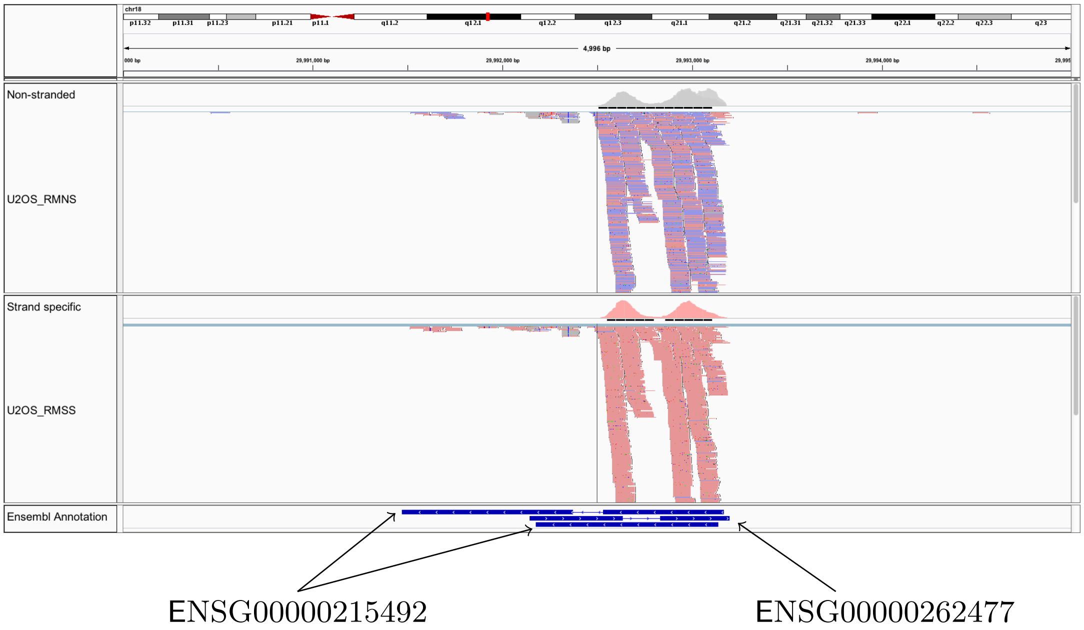

Non-coding gene found to be significantly higher expressed in the strand specific data compared to the non-stranded data. This gene, ENSG00000215492, is a pseudogene and has two annotated isoforms, ENST00000400448 and ENST00000521273, shown at the annotation track at the bottom of the figure. The annotation also shows an overlapping antisense gene, ENSG00000262477, which is a lincRNA. All reads that map to any overlapping exons of these two genes are labelled as ambiguous for the non-stranded data (top coverage track) hence its low expression as determined by htseq-count. For the stranded data, however, htseq-count has no problem of assigning the reads to the overlapping genes. This scenario appears to be dominant in explaining why genes get labelled as being significantly higher expressed in the stranded data compared to the non-stranded data.

C Unannotated overlapping feature. Chr17:66107000-66129000

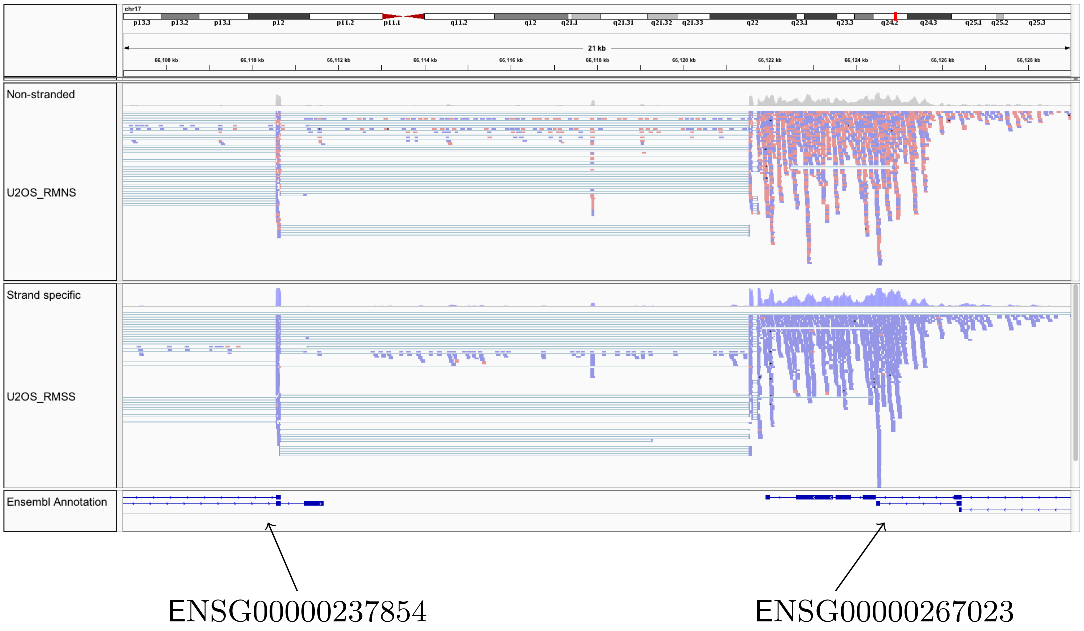

Non-coding gene found to be significantly higher expressed in the non-stranded data compared to the strand specific data. This gene, ENSG00000267023, is a pseudogene and has three annotated isoforms, shown at the annotation track at the bottom of the figure. The annotation also shows another gene, ENSG00000237854 to the left. These two genes, ENSG00000267023 and ENSG00000237854, while not annotated os overlapping are in the opposite directions. For the strand specific data, shown on the bottom coverage track, blue reads map to annotations in the right direction as can be seen from the gene on the left. The pseudogene, however, is annotated in the left direction and since no reads map to that strand (all reads are blue) htseq-count does not assign any reads to this feature. For the non-stranded data, however, htseq-count assigns all the reads in this loci to this feature. The fault here lies in the annotation of the gene, ENSG00000237854, which, according to this coverage plot, should have an isoform extending to at least 66.126 kb. Indeed, the RefSeq annotation (not shown here) has such an isoform extending to 66.132 kb.

D Unannotated overlapping feature. Chr8:42977000-42988000

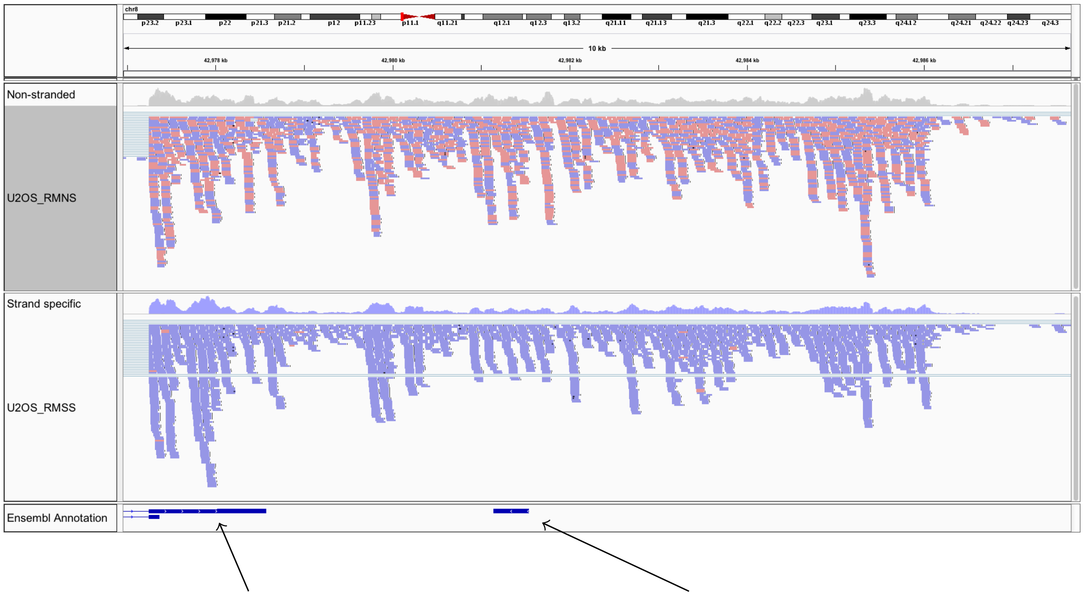

ENSG00000185900 ENSG00000254198

Non-coding gene found to be significantly higher expressed in the non-stranded data compared to the strand specific data. This gene, ENSG00000254198, is a pseudogene and has one annotated isoform shown at the annotation track at the bottom of the figure. The annotation also shows a protein coding gene, ENSG00000185900 to the left. These two genes, ENSG00000254198 and ENSG00000185900, while not annotated os overlapping are in the opposite directions. For the stranded data, shown on the bottom coverage track, blue reads map to annotations in the right direction as can be seen from the protein coding gene on the left. The non-coding gene, however, is annotated in the left direction and since no reads map to that strand (all reads are blue) htseq-count does not assign any reads to this feature. For the non-stranded data, however, htseq-count assigns all the reads in this loci to this feature. The fault here lies in the annotation of the protein coding gene, ENSG00000185900, which, according to this coverage plot, should extend to 42.986 kb.

E

Annotation in the wrong direction.
Chr10:52413000-52423000

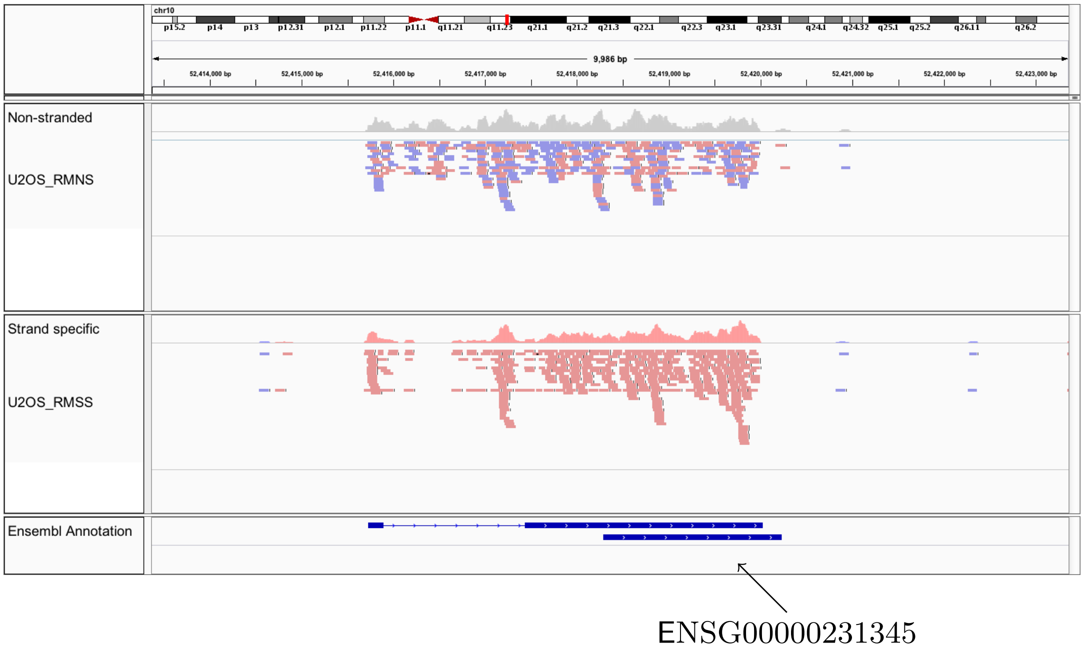

Non-coding gene found to be significantly higher expressed in the non-stranded data compared to the strand specific data. This gene, ENSG00000231345, is a pseudogene and has two annotated isoforms shown at the annotation track at the bottom of the figure. Both coverage tracks, the non-stranded at the top and the stranded below, show expression at this loci but all the stranded reads are to the left (red) and the annotation is to the right. That is why htseq-count assigns no reads to this feature for the stranded data but does so for the non-stranded data. Likely explanation for this is that the gene is annotated in the wrong direction.

F Annotation in the wrong direction.
ChrX:139864000-139869000

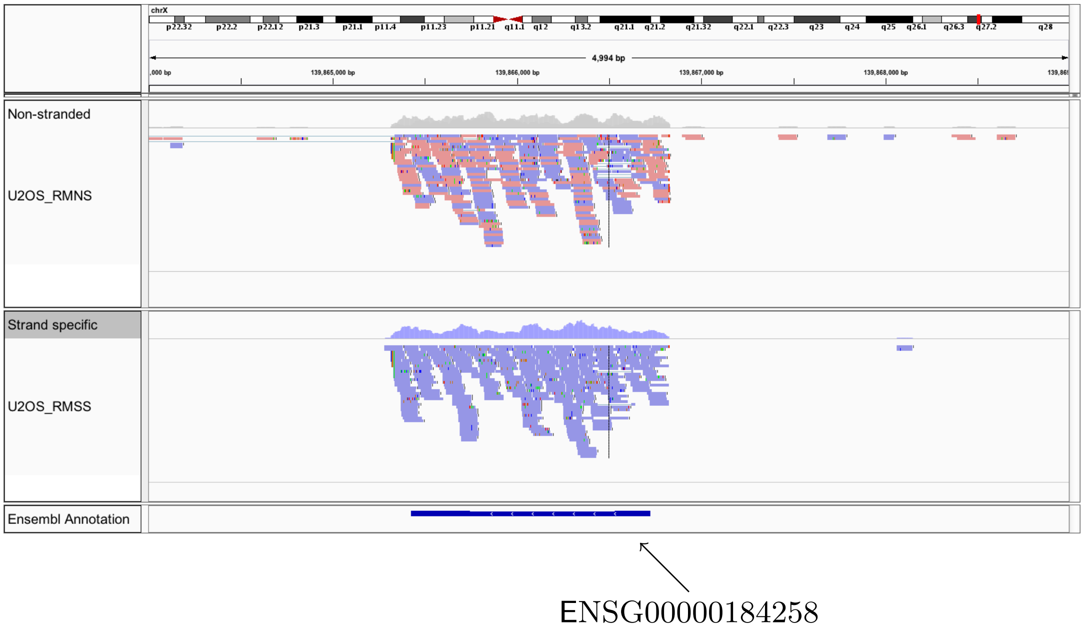

Protein coding gene found to be significantly higher expressed in the non-stranded data compared to the strand specific data. This gene, ENSG00000184258, has only one isoform shown at the annotation track at the bottom of the figure. Both coverage tracks, the non-stranded at the top and the strand specific below, show expression at this loci but all the stranded reads are to the right (blue) and the annotation is to the left. That is why htseq-count assigns no reads to this feature for the strand specific data but does so for the non-stranded data. Likely explanation for this is that the gene is annotated in the wrong direction.

G Antisense intronic transcripts.
Chr7:102270000-102310000

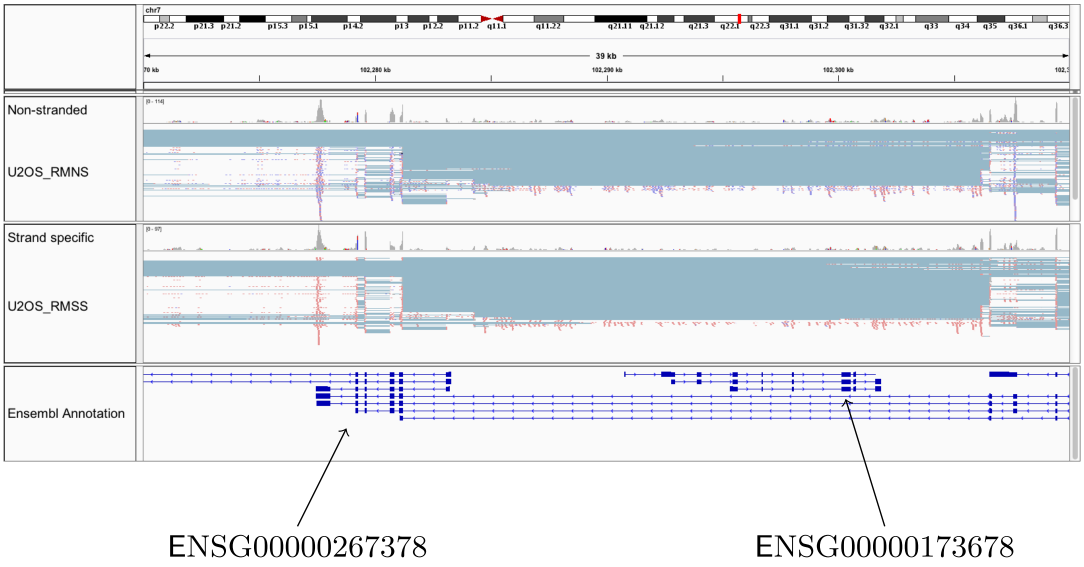

Protein coding gene found to be significantly higher expressed in the non-stranded data compared to the strand specific data. This gene, ENSG00000173678, has three isoforms shown at the annotation track at the bottom of the figure. As can be seen from the annotation track, this gene is opposite to an intron of another gene, ENSG00000267378. Looking at the stranded data it can be seen that all the reads come from this intron (red reads, left direction) and thus no reads get mapped to ENSG00000173678. For the non-stranded data, however, all the reads (both blue and red) get assigned to ENSG00000173678 and hence the higher expression of that gene in the non-stranded data.

H Antisense intronic transcripts. Chr11:57403000-57423000

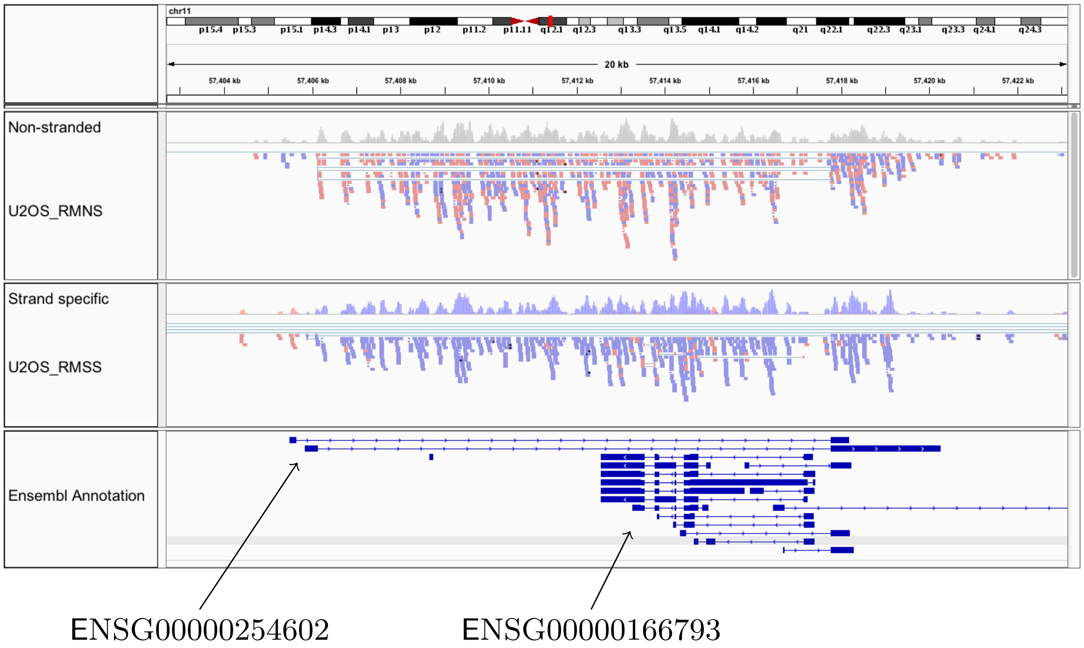

Protein coding gene found to be significantly higher expressed in the non-stranded data compared to the strand specific data. This gene, ENSG00000166793, has 11 isoforms shown at the annotation track at the bottom of the figure. As can be seen from the annotation track, this gene is opposite to an intron of the gene ENSG00000254602. Looking at the stranded data it can be seen that nearly all the reads come from this intron (blue reads, right direction) and thus very few reads get mapped to ENSG00000166793. For the non-stranded data, however, all the reads (both blue and red) get assigned to ENSG00000166793 and hence the higher expression of that gene in the non-stranded data.
