# Supplementary material for: Analysis of stranded information using an automated procedure for strand specific RNA sequencing
Source: BMC Genomics. 2014 Jul 28;15(1):631. doi: 10.1186/1471-2164-15-631 (PMC4247151; doi:10.1186/1471-2164-15-631)
Supplement: Supplementary file 9 — Additional file 9: Figure S5. Coverage plot showing that the novel transcription shown in Figure 5 in the main text is exclusive to the U2OS cell line. (PDF 91 KB) [file 12864_2014_6674_MOESM9_ESM.pdf]

Analysis of stranded information using an automated procedure for strand specific RNA sequencing

Additional file 9

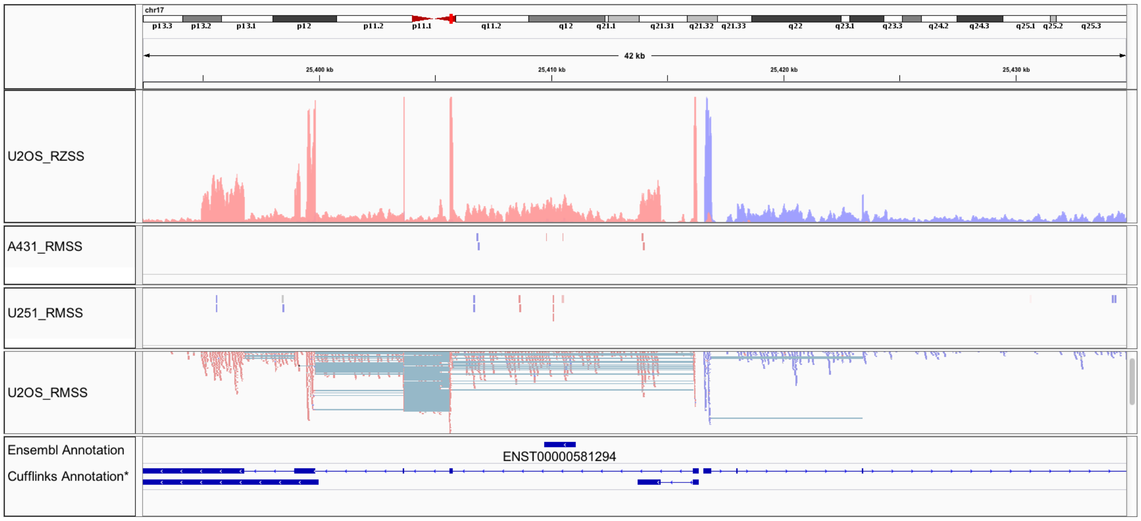

Figure S5: Novel gene on chromosome 17 shows cell specific transcription. Comparison of mapped data from libraries 1-8; A431_SSRM, U251_RMSS and U2OS_RMSS. The cell lines A431 and U251 show virtually no expression in this loci, but the U2OS cell line does.
