# Supplementary material for: Analysis of stranded information using an automated procedure for strand specific RNA sequencing
Source: BMC Genomics. 2014 Jul 28;15(1):631. doi: 10.1186/1471-2164-15-631 (PMC4247151; doi:10.1186/1471-2164-15-631)
Supplement: Supplementary file 10 — Additional file 10: Figure S6. Another novel transcription exclusive to the U2OS cell line. (PDF 80 KB) [file 12864_2014_6674_MOESM10_ESM.pdf]

Analysis of stranded information using an automated procedure for strand specific RNA sequencing

Additional file 10

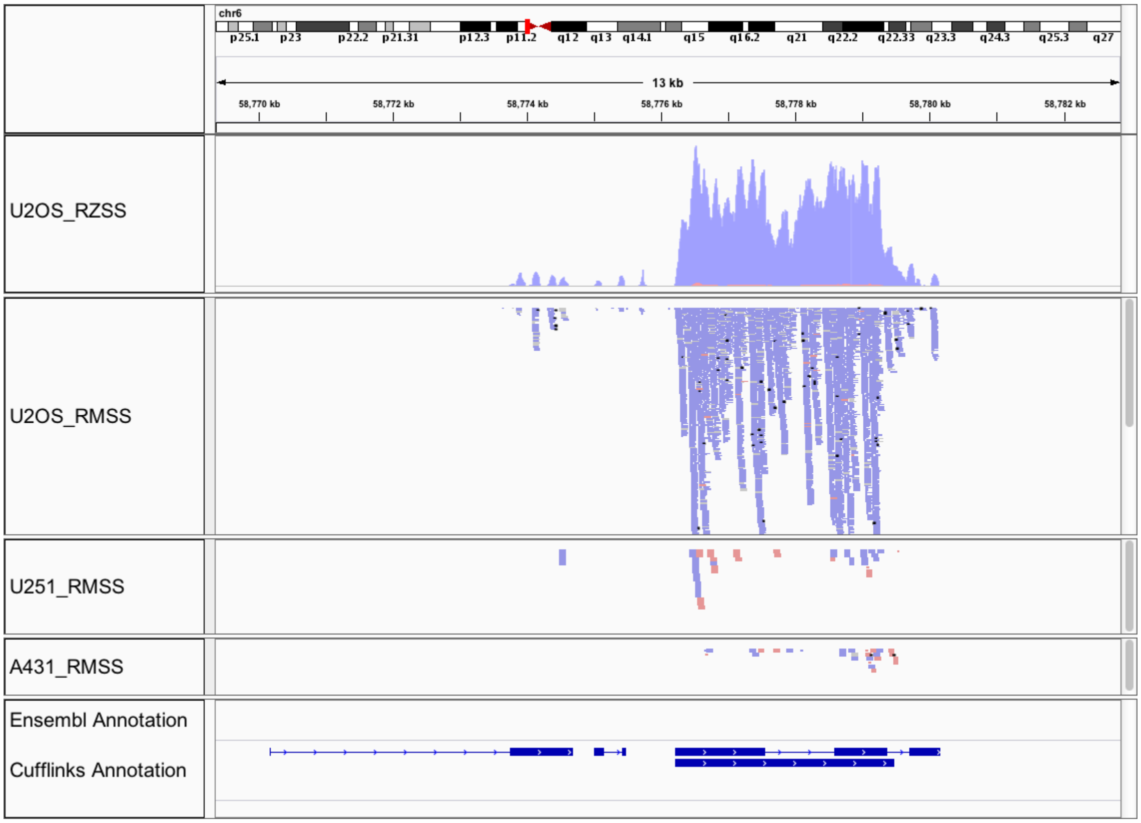

Figure S6: Novel gene on chromosome 6 shows cell specific transcription. The U2OS cell line is actively transcribed in this loci, which is unannotaded in Ensembl. The U251 cell line and the A431 cell line show very little expression in this lici compared to the U2OS cell line.
