# Supplementary material for: Analysis of stranded information using an automated procedure for strand specific RNA sequencing
Source: BMC Genomics. 2014 Jul 28;15(1):631. doi: 10.1186/1471-2164-15-631 (PMC4247151; doi:10.1186/1471-2164-15-631)
Supplement: Supplementary file 11 — Additional file 11: Figure S7. Coverage plot indicating that two exons annotated as two genes may actually be two exons from the same gene. (PDF 91 KB) [file 12864_2014_6674_MOESM11_ESM.pdf]

Analysis of stranded information using an automated procedure for strand specific RNA sequencing

Additional file 11

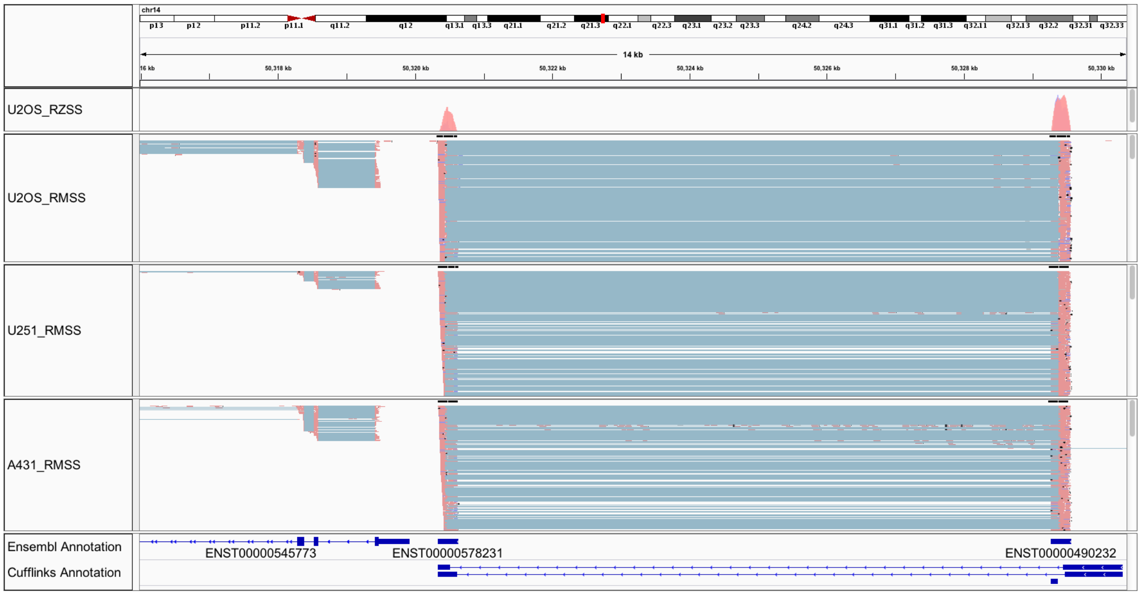

Figure S7: Ubiquitous transcription on chromosome 14 suggests fault in current annotation. As shown in the annotation tracks at the bottom of the figure, this loci is annotated as two genes in Ensembl (ENST00000578231 and ENST00000490232). Our data suggests that these are two exons of the same gene as shown by the inserts in the read data and the Cufflinks annotation track.
