# Supplementary material for: Analysis of stranded information using an automated procedure for strand specific RNA sequencing
Source: BMC Genomics. 2014 Jul 28;15(1):631. doi: 10.1186/1471-2164-15-631 (PMC4247151; doi:10.1186/1471-2164-15-631)
Supplement: Supplementary file 12 — Additional file 12: Figure S8. The raw transcript assembly around the PTENP1 locus highlighting the manual changes made for the proposed assembly. (PDF 133 KB) [file 12864_2014_6674_MOESM12_ESM.pdf]

Analysis of stranded information using an automated procedure for strand specific RNA sequencing

Additional file 12

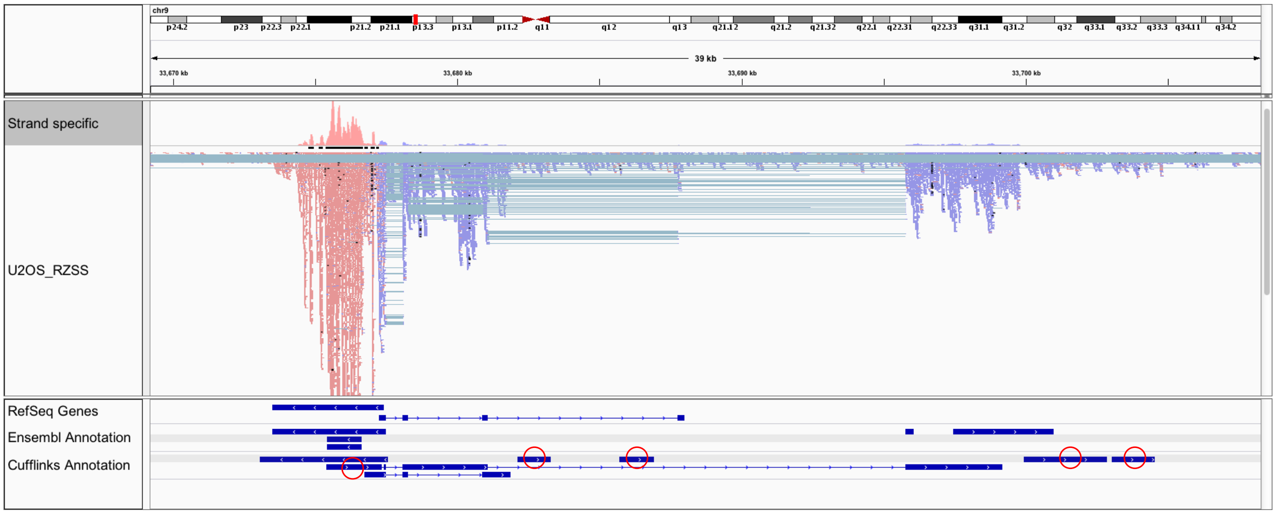

Figure S8: The Cufflinks assembly shown in Figure 4 is not the direct output from Cufflinks but has been cleaned up manually. The loci that have been cleaned up are marked with red circles; the two exons close to the first circle were merged into one exon and shortened, the other circles show exons that were removed from the assembly.
