# Supplementary material for: Analysis of stranded information using an automated procedure for strand specific RNA sequencing
Source: BMC Genomics. 2014 Jul 28;15(1):631. doi: 10.1186/1471-2164-15-631 (PMC4247151; doi:10.1186/1471-2164-15-631)
Supplement: Supplementary file 13 — Additional file 13: Figure S9. The raw transcript assembly around the locus of the novel gene in Figure 5 highlighting the manual changes made for the proposed assembly. (PDF 92 KB) [file 12864_2014_6674_MOESM13_ESM.pdf]

Analysis of stranded information using an automated procedure for strand specific RNA sequencing

Additional file 13

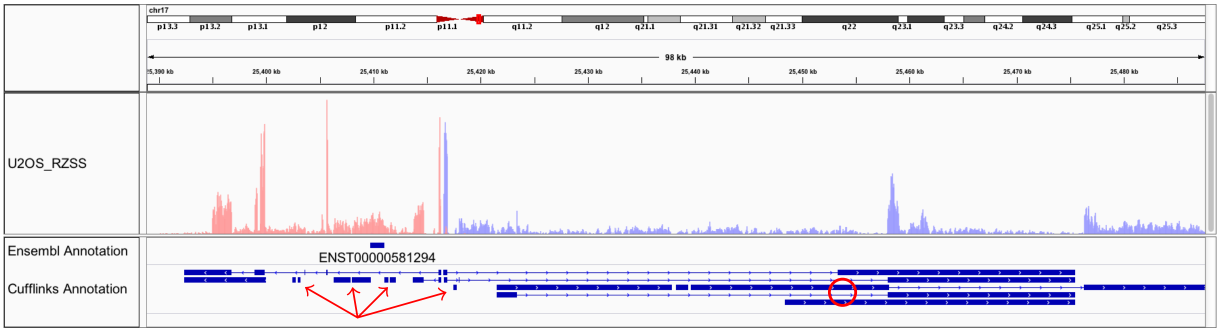

Figure S9: The Cufflinks assembly shown in Figure 5 is not the direct output from Cufflinks but has been cleaned up manually. This figure shows the raw output from Cufflinks. The loci that have been removed in Figure 5 are marked with red arrows (small transcripts) and a red circle.
