# Supplementary material for: Analysis of stranded information using an automated procedure for strand specific RNA sequencing
Source: BMC Genomics. 2014 Jul 28;15(1):631. doi: 10.1186/1471-2164-15-631 (PMC4247151; doi:10.1186/1471-2164-15-631)
Supplement: Supplementary file 14 — Additional file 14: Supplementary file S1. File listing some of the command lines used for the analysis. (PDF 87 KB) [file 12864_2014_6674_MOESM14_ESM.pdf]

Analysis of stranded information using an automated procedure for strand specific RNA sequencing

Additional file 14

These are some examples of the command lines used in this study. Sometimes, directory paths have been omitted for clarity. This list is not exhaustive and for specific information on command line usage in this study please contact:
benjamin.sigurgeirsson@scilifelab.se

TrimGalore

```
$ trim_galore -a GATCGGAAGAGCACACGTCTGAACTCCAGTCACGTGAAACGATCTCGTATGCCGTCTTCTGCTTG
-q 20 --stringency 5 --paired --length 20 U2OS_RZ_R4_1.fastq.gz U2OS_RZ_R4_2.fastq.gz
```

Tophat2

```
$ tophat -o [path to output directory] --library-type fr-firststrand
--solexa1.3-quals -p 8 --rg-platform illumina --rg-platform-unit 1 --rg-center
SciLife --GTF ensembl72.gtf hg19_primary U251_SS_R1_1.fastq U251_SS_R1_2.fastq
```

Star: index generation

```
$ STAR --runMode genomeGenerate --genomeDir [path to index directory]
--genomeFastaFiles hg19_primary.fa --runThreadN 8
```

Star: read alignment

```
$ STAR --genomeDir [path to index directory] --runThreadN 8 --readFilesIn
U2OS_RZ_R1_1.fastq U2OS_RZ_R1_2.fastq --outReadsUnmapped Fastx --chimSegmentMin
15 --chimJunctionOverhangMin 15 --outFileNamePrefix [path to output directory]
```

Samtools: filter out non-primary alignments

```
$ samtools view -F 256 -b accepted_hits.bam > primary_hits.bam
```

RSeQC: filter out ribosomal reads

```
$ python split_bam.py -i primary_hits.bam -r rRNA.bed -o rRNA
```

RSeQC: filter out reads mapping outside of annotation

```
$ python split_bam.py -i rRNA.ex.bam -r ensembl.bed -o novel
```

htseq-count

```
$ python htseq-count -m union -s reverse RZR1.rRNA.ex.sam ensembl72.gtf >
RZR1.count
```

Cufflinks: assembly of all reads

```
$ cufflinks -o [path to output directory] -F 0.1 -j 0.15 -p 8 --library-type
fr-firststrand RZR1.rRNA.ex.bam
```

Cufflinks: assembly of novel/unannotated reads

```
$ cufflinks -o [path to output directory] -F 0.1 -j 0.15 -p 8 -M ensembl72.gtf
--library-type fr-firststrand RZR1.novel.ex.bam
```
